# A WNT mimetic with broad spectrum FZD-specificity decreases fibrosis and improves function in a pulmonary damage model

**DOI:** 10.1186/s12931-024-02786-2

**Published:** 2024-04-02

**Authors:** Mehaben Patel, Yorick Post, Natalie Hill, Asmiti Sura, Jay Ye, Trevor Fisher, Nicholas Suen, Mengrui Zhang, Leona Cheng, Ariel Pribluda, Hui Chen, Wen-Chen Yeh, Yang Li, Hélène Baribault, Russell B. Fletcher

**Affiliations:** Surrozen, Inc., 171 Oyster Point Blvd, Suite 400, South San Francisco, CA 94080 USA

**Keywords:** WNT mimetic, Wnt signaling, Pulmonary fibrosis, IPF, AT2 cell, AT2 organoid, Alveolosphere

## Abstract

**Background:**

Wnt/β-catenin signaling is critical for lung development and AT2 stem cell maintenance in adults, but excessive pathway activation has been associated with pulmonary fibrosis, both in animal models and human diseases such as idiopathic pulmonary fibrosis (IPF). IPF is a detrimental interstitial lung disease, and although two approved drugs limit functional decline, transplantation is the only treatment that extends survival, highlighting the need for regenerative therapies.

**Methods:**

Using our antibody-based platform of Wnt/β-catenin modulators, we investigated the ability of a pathway antagonist and pathway activators to reduce pulmonary fibrosis in the acute bleomycin model, and we tested the ability of a WNT mimetic to affect alveolar organoid cultures.

**Results:**

A WNT mimetic agonist with broad FZD-binding specificity (FZD1,2,5,7,8) potently expanded alveolar organoids. Upon therapeutic dosing, a broad FZD-binding specific Wnt mimetic decreased pulmonary inflammation and fibrosis and increased lung function in the bleomycin model, and it impacted multiple lung cell types *in vivo.*

**Conclusions:**

Our results highlight the unexpected capacity of a WNT mimetic to effect tissue repair after lung damage and support the continued development of Wnt/β-catenin pathway modulation for the treatment of pulmonary fibrosis.

**Supplementary Information:**

The online version contains supplementary material available at 10.1186/s12931-024-02786-2.

## Background

Idiopathic pulmonary fibrosis (IPF) is an interstitial lung disease characterized by areas of myofibroblast accumulation and extracellular matrix (ECM) deposition, disruption of alveolar architecture, and restricted lung physiology [1–3]. The median survival time after diagnosis is 2 to 5 years [4]. Anti-fibrotic agents Pirfenidone and Nintedanib, approved by the United States Food and Drug Administration in 2014, slow the rate of pulmonary functional deterioration [5–8]. However, lung transplantation remains the only therapeutic option that prolongs life expectancy for patients with IPF [9, 10], highlighting the need for new approaches.

According to the predominant pathogenesis hypothesis, injured distal airway and alveolar epithelial cells initiate the disease process, ultimately leading to myofibroblast activation and excessive ECM production [11–14]. In support of this model, damage to AT2 cells has been found to induce fibrosis [15]. Moreover, *Sftpc* and telomere related mutations in AT2 cells have been found to drive fibrosis in animal models and to be associated with pulmonary fibrosis in humans [16–20]. More recent scRNA-seq and functional studies have shown that aberrant epithelial cells are a central feature of pulmonary fibrosis in humans and in animal models and have pro-fibrotic activity [21–30].

The canonical Wnt/β-catenin signaling pathway plays a constructive role in the lung alveolar epithelium. During development this pathway is critical for the establishment of alveoli [31–33]. In adult lungs, a subset of alveolar type 2 (AT2) cells are stem cells [34–38], and Wnt/β-catenin signaling is required for AT2 cell maintenance and renewal under both normal and injured conditions [36, 37].

In contrast, over the past 20 years many studies have reported that active Wnt/β-catenin signaling promotes pulmonary fibrosis, predominantly by affecting stromal and epithelial cells. An accumulation of nuclear/activated β-catenin in or near fibrotic foci and elevated expression of Wnt target genes have been observed in mouse models of pulmonary fibrosis and IPF patient samples [39–43]. Overexpression of siRNA targeting β-catenin (*Ctnnb1*) or administration of small molecules (ICG-001, XAV-939) that interfere with Wnt signaling decreases fibrosis in animal models [44–49], and germline loss of function mutations in *Fzd8* or *Lrp5* have demonstrated resistance to bleomycin-induced fibrosis [50, 51]. Conversely, activation of Wnt signaling in pulmonary capillary endothelial cells promotes perivascular fibroblast activation and fibrosis [52], and WNT3A and WNT7A have pro-fibrogenic activity in fibroblast cell lines [42, 53]. Finally, the ability of TGFβ to induce myofibroblast activation has been reported to depend on Wnt/β-catenin signaling [43, 48, 54].

WNT ligands bind to FZD and LRP receptors to initiate canonical Wnt/β-catenin signaling, and RSPO potentiates this signaling by removing E3 ubiquitin ligases from the surface that normally target WNT receptors for degradation [55]. Potent antibody-based modulators of Wnt signaling have recently been developed [56–60], and treatment with WNT or RSPO mimetics can promote tissue repair in *in vivo* models of bone, colon, liver, and ocular damage [57, 61–64]. We found that a multi-FZD-specific WNT mimetic agonist led to expansion of alveolar cells *in vitro*. Due to the potentially opposing roles of Wnt signaling in promoting alveolar regeneration and pulmonary fibrosis, we tested the effects of antibody-based positive and negative modulators of Wnt signaling in an acute bleomycin mouse model of pulmonary fibrosis. In a therapeutic dosing regimen, activation of Wnt/β-catenin signaling in the lungs with a multi-FZD-specific WNT mimetic decreased fibrosis and improved lung function. Our work unexpectedly indicated that transient activation of the Wnt/β-catenin pathway may have promise in mitigating pulmonary fibrosis.

## Methods

### Animals

All animal experimentation was in accordance with the criteria of the *Guide for the Care and Use of Laboratory Animals*, 8th edition, as prepared by the National Academy of Sciences. Protocols for animal experimentation were approved by the Surrozen Operating, Inc. Institutional Animal Care and Use Committee (IACUC). Upon arrival, mice were housed using the Innovive Disposable IVC Rodent Caging System for mice (Innovive, San Diego, CA), and acclimatized for a minimum of two days prior to the start of the study. Mice were given ad libitum access to food (2018 Teklad global 18% protein rodent diet, Envigo, Indianapolis, IN) and purified, laboratory-grade acidified water.

Termination was conducted in compliance with the current requirements of *Guide for the Care and Use of Laboratory Animals*, 8th edition, and the American Veterinary Medical Association (AVMA) Guidelines on Euthanasia.

### *In vivo* studies and tissue collection

#### Study #1, Agonist vs. antagonist

13-weeks old male C57BL/6J mice (Jackson Laboratories) were assigned to saline control (*n* = 10) or dosing with bleomycin (*n* = 36). Saline or Bleomycin (0.25 mg/kg) was administered on Day 0 intratracheally in a 50 µL volume followed by 50 µL of air to ensure uniform distribution of bleomycin in alveoli. From bleomycin-treated mice, 10 mice were excluded from the study prior to randomization on day 7, either due to body weight loss and for compliance with humane endpoints, or due to less than 5% body weight loss, which reflected low or no bleomycin-induced injury. On day 7, the remaining 26 mice were divided into 3 groups and were injected intraperitoneally with a bi-weekly dose (day 7, 10, 14, 17) of R2M3 (10 mg/kg, *n* = 9) or anti-green fluorescent protein (Anti-GFP) (10 mg/kg, *n* = 8) or R2M3-26 + RSPO2 (Combo) (1 + 0.3 mg/kg, *n* = 9) groups for two weeks. Mice were terminated on day 21. Body weight was measured on a daily basis. At termination on day 21, mice were anesthetized with isoflurane, followed by cervical dislocation. Lung lobes were perfused with PBS. The needle tip was inserted into the trachea and held in place with tied suture. The lung was inflated gently until all lobes were fully, uniformly, and consistently expanded. The perfused left lung lobes were immediately fixed in 10% neutral buffered formalin and moved to 70% Ethanol after 24 h until embedding. After embedding and mounting of the tissue, the slides were stained with Hematoxylin and Eosin (H&E) and Masson’s Trichome. Additional lung lobe samples were snap-frozen in liquid nitrogen and stored at -80 ºC for gene expression analyses.

#### Study #2, WNT mimetic vs. RSPO2

13-weeks old male C57BL/6J mice (Jackson Laboratories) were assigned to saline control (*n* = 5) or dosing with bleomycin (*n* = 59). Saline or Bleomycin (0.25 mg/kg) was administered on Day 0 intratracheally in a 50 µL volume followed by 50 µL of air to ensure uniform distribution of bleomycin in alveoli. From bleomycin treated mice, 16 were excluded prior to randomization due to body weight loss, as in study #1. On day 7, the remaining 43 mice were divided into 5 groups and were injected intraperitoneally with a bi-weekly dose (day 7, 10, 14, 17) of R2M3-26 (10 mg/kg, *n* = 9) or anti-green fluorescent protein (Anti-GFP) (10 mg/kg, *n* = 9) or R2M3-26 + RSPO2 (Combo) (1 + 0.3 mg/kg, *n* = 7) or RSPO2 (1 mg/kg, *n* = 9) or RSPO2 (4.6 mg/kg, *n* = 9) groups for two weeks. Mice were terminated on day 21. Body weight was measured on a daily basis. Tissue collection was the same as study #1.

#### Study #3, WNT mimetic functional study

Study 3 was performed by IPS Therapeutique, Inc. in Sherbrooke Québec. 10–12 weeks old male C57BL/6J mice (Charles River Laboratories) were assigned to saline control (*n* = 10) or dosing with bleomycin (*n* = 24). Animals from bleomycin group received intratracheal instillation of 2 mg/kg of bleomycin solution on Day 0 and Day 7. Animals from the saline group received intratracheal instillation of 0.9% saline solution on Day 0 and Day 7. Four mice from the bleomycin were removed from the study during randomization on day 10. After randomization, remaining 20 mice were divided into two groups (*n* = 10/group) and were injected intraperitoneally with either R2M3-26 (30 mg/kg) or Anti-Green Fluorescent protein (Anti-GFP) (30 mg/kg) twice a week for 2.5 weeks (on days 10, 14, 17, 21 and 24). Tissue samples were collected at termination, on Day 28.

Three days before the bleomycin instillation, all mice were acclimated to the plethysmograph chamber for 10 min per day. On Day 0 all mice were introduced to the plethysmograph chamber environment. The functional respiratory parameters were assessed by the whole-body plethysmograph (VivoFlow, SCIREQ) on Day 28 post-bleomycin instillations. Body weight was monitored from Day 0 to Day 10 and after bi- weekly. Mice were terminated on day 28.

The left lung was used for the histology analyses. The pulmonary airway was flushed with 0.9% NaCl and the left lobe was inflated using a syringe filled with a fixative (10% NBF) with attached blunt tip needle. The needle tip was inserted into the trachea and held in place with tied suture. The lung was inflated gently at a physiological pulmonary pressure until all lobes were fully, uniformly, and consistently expanded. The tissues were kept in formalin for 48 h, and then transferred in PBS at 4 °C until shipment. After embedding and mounting of the tissue, the slides were stained with Hematoxylin and Eosin (H&E), Masson’s Trichome and with PicroSirius red.

To calculate the lung index, the chest cavity was further opened to expose the lungs, which were then excised and weighted wet to determine the lung weight and lung index. The right lung was tied off and collected immediately, separating the four lobes, weighed, snap-frozen into liquid nitrogen and stored at -80 °C.

### Respiratory functional test

Mice were anaesthetized with a mixture of 2.5% isoflurane. Mice were immediately ventilated by means of a FlexiVent. Following stabilization, all mice were submitted to Mouse Mechanic Scan script protocol, including SnapShot-150, Quickprime-3, PV Loop and Deep inflation ventilatory pattern protocols, which allows the calculation of the inspiratory capacity (IC), elastance, and compliance of the respiratory system. The lungs were initially inflated to 30 cmH_2_O and then allowed to return to resting ventilation volume. This brief pause between pressure increments allowed for equilibration of pressure, allowing for static measurement in pulmonary function. Following the recording, mice were euthanized by exsanguination. The whole blood was collected and divided to serum and plasma and stored at − 80 °C.

### BALF collection for cytokine level

The thoracic cavity was opened to expose the lungs. The left lung was clamped while 0.9 mL of a cold solution of PBS 1X, Protease Inhibitor 1 × (3 x 300 µL) was injected by the trachea to perform a bronchoalveolar lavage (BAL) on the right lobe of the lungs. The BAL fluid (BALF) was separated into aliquots, snap-frozen and kept at -80 °C for analysis of cytokine levels (MD44 panel).

### Collagen quantification

The middle lobe was used for quantification of the total soluble lung collagen. The lung was homogenized using a polytron on ice, in PBS 1X + 0.1% Triton X-100 with protease cocktail inhibitors to obtain a homogenate. The homogenate was then centrifuged at 1500 rpm for 15 min at 4 °C. An aliquot of the lung homogenate was thawed on ice and transferred to a low retention microtube for soluble collagen quantification, using the Sircol Soluble Collagen Assay (Biocolor, S1111).

### Histopathology assessment

For all three *in vivo* animal studies whole slide scans of the Hematoxylin and Eosin (H&E), Masson’s Trichome, and PicroSirius red stained sections of lung tissue were imaged. Histology-stained samples were manually scored in a blinded manner by a board-certified veterinary pathologist (Vincent Meador, DVM, ACVP, at Pacific Tox Path, LLC) for a range of pathological metrics (see Supplementary Table 1), culminating in the scores presented in the paper, Severity Score (sum of the individual metrics), Percentage Lung Affected, Modified Ashcroft Score.

### Fluorescent mRNA in situ hybridization, immunofluorescence

For immunofluorescence (IF), slides with 5 μm thick formalin fixed paraffin embedded tissue sections were deparaffinized followed by citrate buffer (pH 6) antigen retrieval for 10 min in a pressure cooker. Slides were then washed in water followed by 1x wash in PBS plus 0.1% Tween-20 (PBST). Subsequently, tissue sections were blocked with serum free protein block (Agilent, X090930-2) for one hour at room temperature before incubation with primary antibodies. Tissue sections were then washed in PBST at least 3 times followed by incubation with fluorescent secondary antibodies. Afterwards, tissue sections were washed with PBST and mounted with Vectashield Vibrance antifade mounting medium with DAPI (Vector Laboratories, H-1800).

Expression of mRNA was detected by RNAscope in situ hybridization (Advanced Cell Diagnostics (ACD)) using the standard RNAscope Fluorescent Reagent Kit v2 protocol and coupled with TSA Plus Cy3. When fluorescent mRNA in situ hybridzation (FISH) was coupled with IF, FISH was performed first and then the IF procedure was followed but without the citrate buffer antigen retrieval.

### Imaging, image analysis

**Fig. 1:**
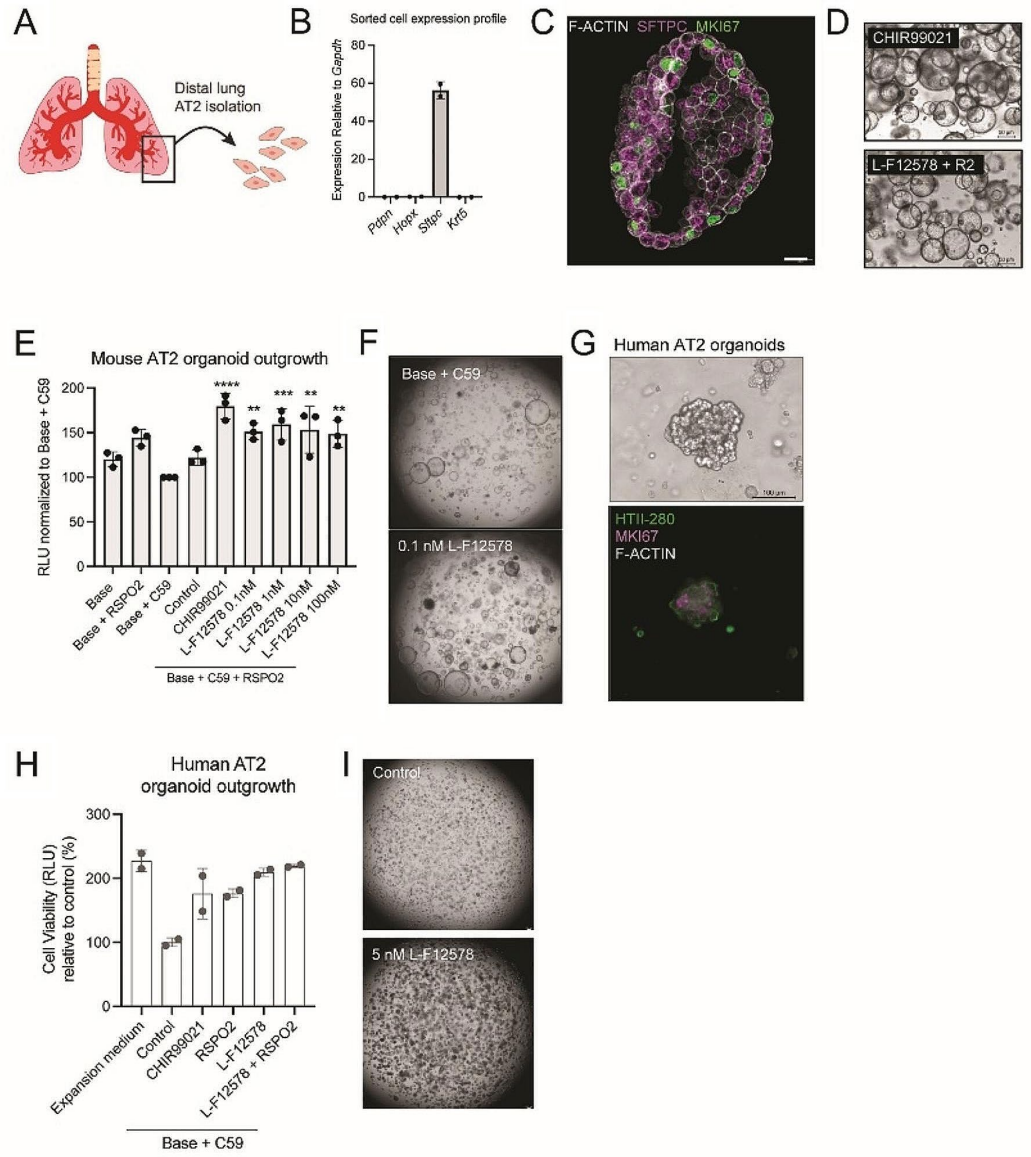
(**A**) Strategy to start AT2 organoid outgrowth from a purified primary cell population. (**B**) Gene expression profile of epithelial lung cell type markers in a population of purified cells used for organoid culture. (**C**) Representative image of mouse AT2 protein expression. Scale bar, 20 μm. (**D**) Mouse AT2 organoids expanded with CHIR99021 or L-F12578 + RSPO2 as a Wnt source. (**E**) Outgrowth efficiency assay of mouse AT2 organoids with various treatments. Statistical analysis: one-way ANOVA, displayed p-value is comparison to Base + C59. **** P value ≤ 0.0001, *** ≤ 0.001, ** ≤ 0.01. (**F**) Representative images of two treatment conditions. (**G**) Representative images of human AT2 organoid morphology and protein expression level after expansion. (**H**) Outgrowth efficiency assay of human AT2 organoids. Wnt mimetic concentration, 5 nM; RSPO2 concentration, 20 nM. (**I**) Representative images at day 12

**Fig. 2:**
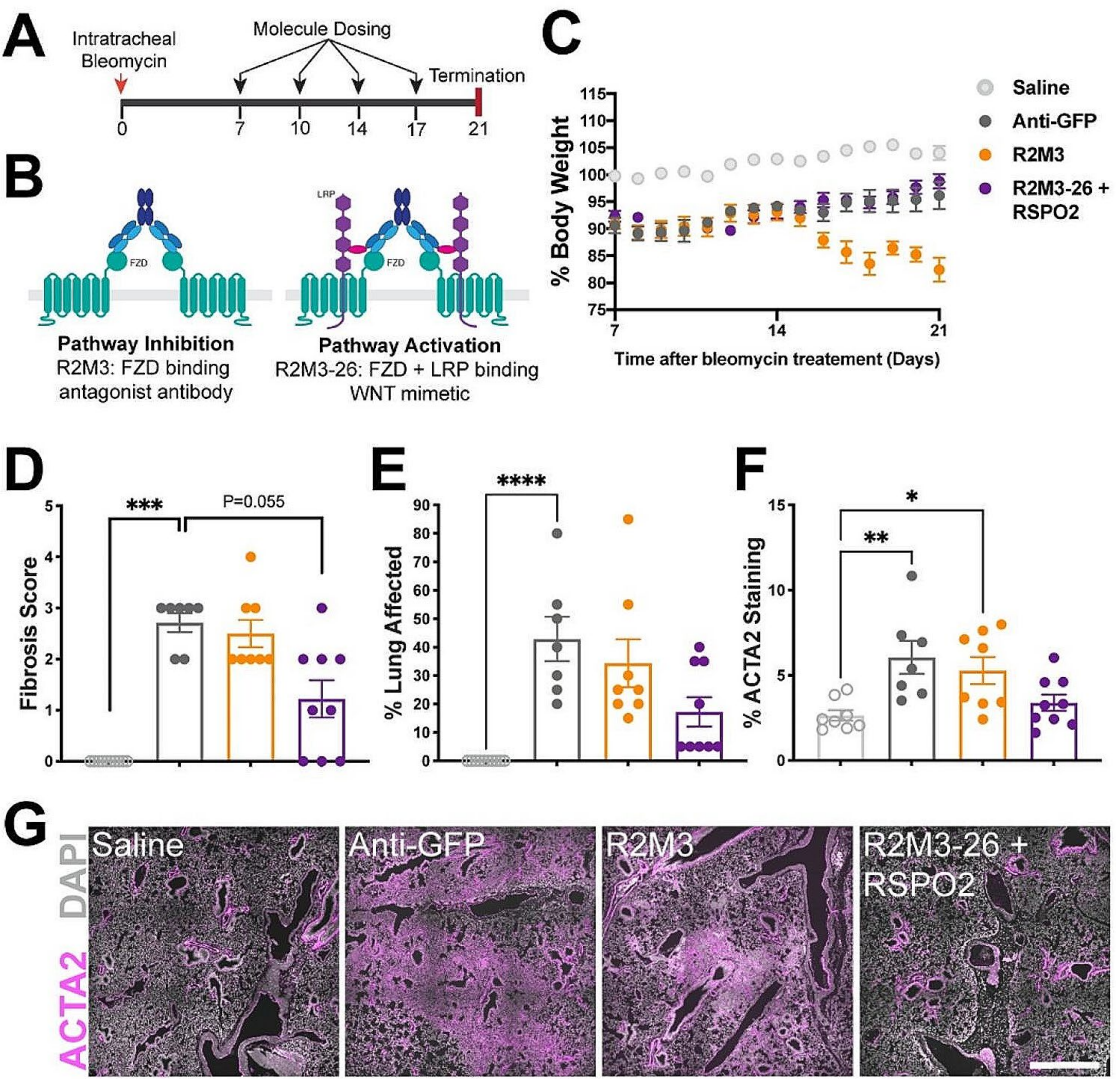
Wnt/β-catenin signaling activation is more beneficial than pathway antagonism in the acute bleomycin model (**A**) Study design, indicating 21-day duration and biweekly dosing starting on day 7 after intratracheal bleomycin administration. (**B**) Schematic representing the test molecules and their receptor interaction: R2M3 only binds and antagonizes FZD receptors, inhibiting the pathway, and the tetravalent, bispecific R2M3-26 WNT mimetic binds FZDs and LRPs and activates the pathway. (**C**) Percentage bodyweight relative to the day 0 value over time. The legend for the treatment groups applies to all plots: Anti-GFP (10 mpk), R2M3 (10 mpk), R2M3-26 (1 mpk) + RSPO2 (0.3 mpk). (**D-E**) Histopathology assessment of the fibrosis score and the percentage lung fibrosis. (**F**) Percentage of the ACTA2 expression area to total tissue area. (**G**) Representative images showing ACTA2 expression for each treatment group; scale bar = 1 mm. Error bars represent SEM; * p value < 0.05, ** < 0.01, *** < 0.001

**Fig. 3:**
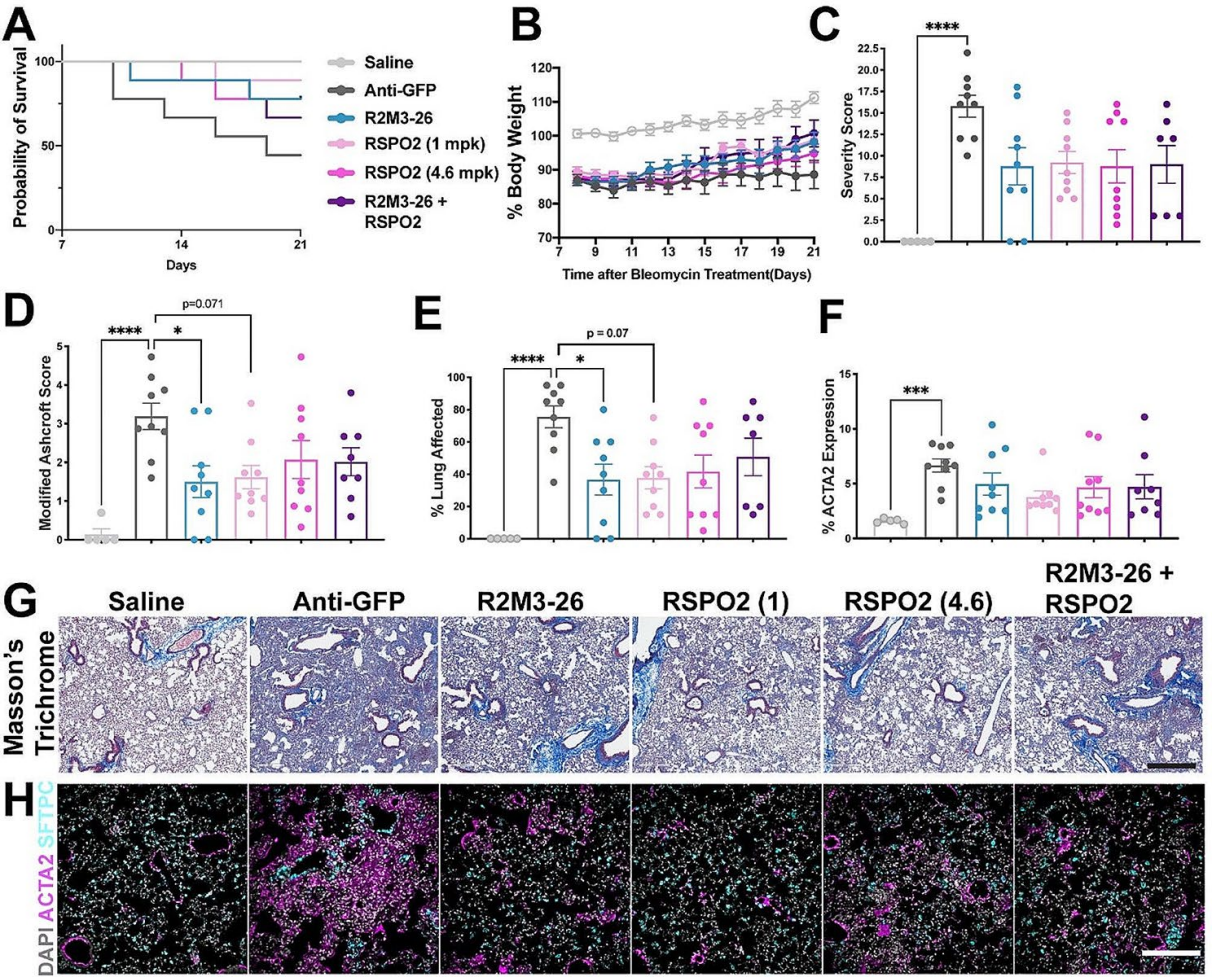
A FZD1,2,5,7,8 targeted WNT mimetic (R2M3-26) is effective in the acute bleomycin model. (**A**) Survival curves by treatment. The treatment legend applies to all graphs in the figure: Saline (uninjured), Anti-GFP (10 mpk), R2M3-26 (10 mpk), RSPO2 (1 mpk), RSPO2 (4.6 mpk), R2M3-26 (1 mpk) + RSPO2 (0.3 mpk). (**B**) Percentage bodyweight relative to day 0 for all groups. (**C-E**) Histopathology assessments. The Severity Score is a composite of multiple histopathology metrics presented in Supplementary Table 1. (**F**) ACTA2 positive area as a percentage of tissue area. (**C-F**) Error bars represent SEM; * p value < 0.05, ** < 0.01, *** < 0.001, **** < 0.0001. (**G**) Representative images of Masson’s trichrome staining in which collagen is blue; scale bar = 1 mm. (**H**) Example IF images of ACTA2 and SFTPC; scale bar = 200 μm

**Fig. 4:**
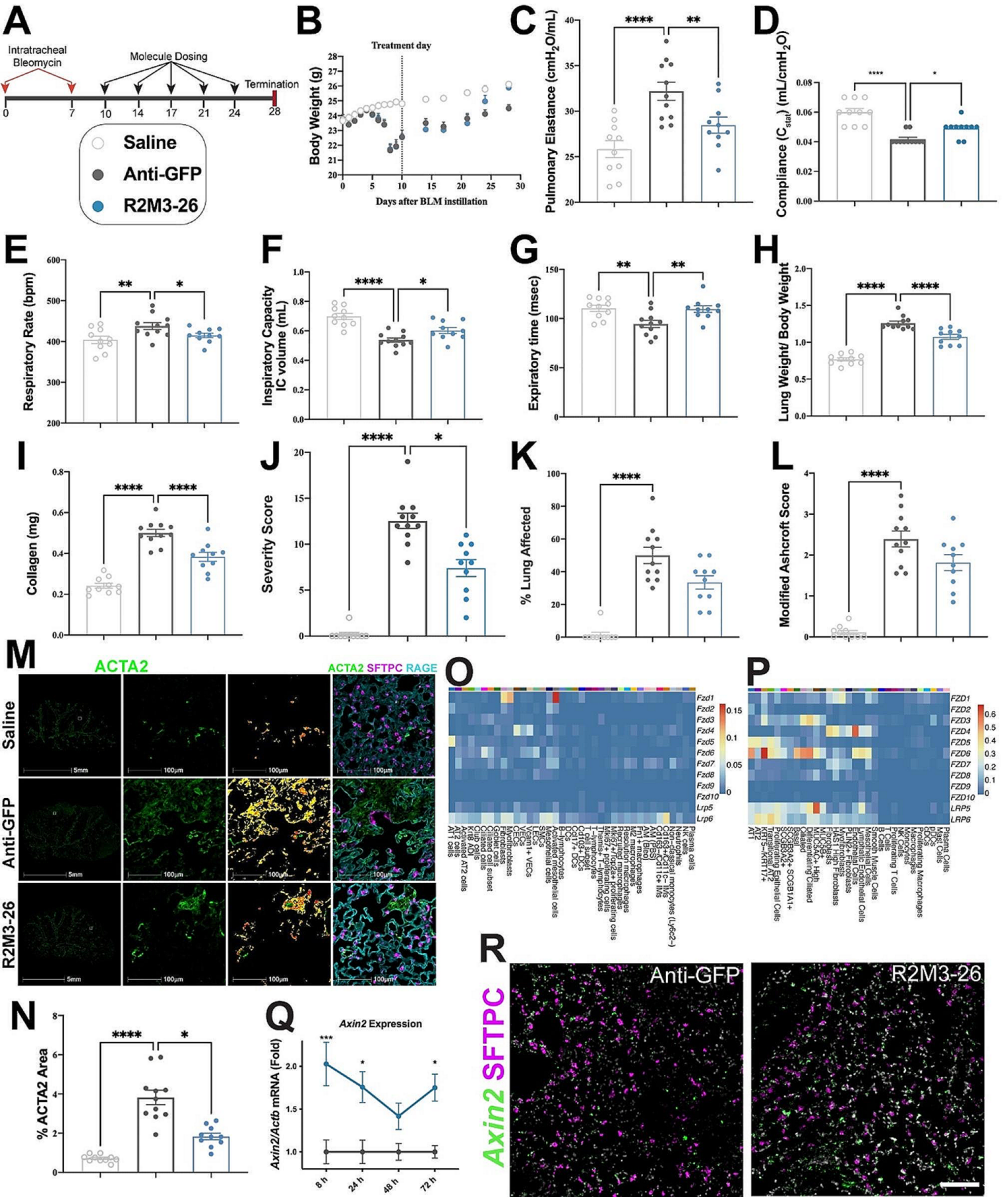
R2M3-26 improves lung function and histology in a two-hit acute bleomycin model. (**A**) Study design showing two-hit bleomycin administration and bi-weekly molecule doses with termination at day 28. Figure legend applies to all graphs, Saline (uninjured), Anti-GFP (30 mpk), R2M3-26 (30 mpk). (**B**) Bodyweight in grams. (**C-G**) Measurements of lung function. (**H**) Ratio of lung weight to bodyweight at termination on day 28. (**I**) Soluble collagen at day 28. (**J-L**) Histopathology assessments. (**M**) Representative IF images showing complete section scans (left column); the three right columns show higher magnification views of the inset box on the left as follows: ACTA2 IF signal, pseudo-colored positive signal levels, and combined ACTA2, SFTPC, and RAGE/AGER IF expression. Each row represents the indicated treatment. (**N**) Percentage of ACTA2 IF signal area to tissue area. (**O**) Average mRNA expression of the *Fzd* and *Lrp* Wnt receptors in mouse scRNA-seq data by cell type from Strunz et al., 2020. (**P**) Average mRNA expression of *FZD* and *LRP* in human scRNA-seq data by cell type from Haberman et al., 2020. (**Q**) *Axin2* expression in the lungs in the acute bleomycin model, assessed by RT-qPCR at the indicated timepoints after a single treatment. (**R**) Representative images of *Axin2* mRNA and SFTPC protein expression at 8 h after a single dose of the indicated treatment; DAPI was used to label nuclei; scale bar = 100 μm. Error bars represent SEM; * p value < 0.05, ** < 0.01, *** < 0.001, **** < 0.0001

Whole slide scans (ZEISS Axioscan 7) were performed on the histology-stained samples for histopathological analysis for all studies. The histology stain images presented in Fig. 1 were obtained on a Lecia DMi-8 microscope equipped with a Leica DFC7000T camera and the fluorescent IF images presented in Figs. 1 and 2 were obtained with the Leica Thunder imaging system. IF images related to Figs. 3 and 4 were obtained with a Zeis Axioscan 7 slide scanner.

Quantification of ACTA2 expression by IF for data presented in Figs. 1 and 2 was on 11–18 10× fields of view obtained on the Lecia Thunder Imaging System. ImageJ was used to apply systematic thresholding and quantify the area of positive signal. For Fig. 3, whole tissue section scans (Zeiss Axioscan 7) of ACTA2 expression were quantified using the HALO IF module, and the AI module was used to remove large airways and airway and vessel lumen regions.

### RNA isolation and RT-qPCR

For RT-qPCR on organoids, RNA was isolated with the Zymo Research Direct-Zol RNA microprep kit (R2660), and the Applied Biosystems Large Capacity cDNA synthesis kit was used for cDNA synthesis. RNA from mouse frozen lung tissues was extracted using the MagMAX™ mirVana™ Total RNA Isolation Kit. cDNA was produced using the high-Capacity cDNA Reverse Transcription Kit or the SuperScript™ IV VILO™ Master Mix. The TaqMan® Fast Advanced Master Mix was used for qPCR. The delta Ct method was used for relative quantification compared to housekeeping genes *Actb* or *Gapdh*.

### Lung tissue dissociation and FACS purification of AT2 cells

Freshly isolated human or mouse lung tissue was washed in cold PBS with 1% antibiotic-antimycotic and any blood clots, mucus, vasculature, and airway structures were removed. Tissue was transferred to a petri dish and minced with scalpel and forceps into fine pieces. Tissue pieces were transferred to a 15 mL conical tube using 12.5 mL of warmed dissociation buffer (DMEM/F12 with Liberase DL 0.3 mg/mL, Dispase II 5 U/mL, DNAase1 50 U/mL, 1% Pen-Strep) and incubated at 37 °C for 30–40 min on a shaker set to 150 rpm. The majority of the solution was filtered through a 100-micron filter into a 50 mL tube containing DMEM/F12 + 10% FBS. The remaining tissue pieces were triturated for 1 min to further dissociate and isolate more cells. The cell suspension was filtered and washed with 12.5 mL of DMEM/F12 + 10% FBS. Cells were centrifuged at 450 rcf for 5 min at 4 °C and supernatant was removed. The pellet was resuspended in 5 mL red blood cell lysis buffer (ACK buffer) and incubated for 3 min at RT. Sample was centrifuged at 450 rcf for 5 min at 4 °C and supernatant was removed. Cells were washed with PBS + 10% FBS, centrifuged at 450 rcf for 5 min at 4 °C and resuspended in FACS buffer (PBS + 2% FBS). After Fc receptor blocking, mouse lung cells were live-dead gated with DAPI, and AT2 cells were FACS purified based on labeling with Anti-EPCAM and high Lysotracker signal. After Fc receptor blocking, human lung cells were live-dead gated with DAPI, and human AT2 cells were FACS purified based on EPCAM + signal, Lysotracker high signal, and HTII-280 + signal.

### Mouse and human alveolar organoid cultures

All mouse and human organoids were maintained at 37 °C in a 5% CO_2_ environment. In this study, sex determination for primary cell cultures was not conducted as it was expected to be of negligible importance. Any sex-related differences in organoid experiments should be further investigated. The following growth conditions were used for feeder-free mouse and human alveolosphere cultures, as described in Katsura et al., 2020.

Mouse alveolar organoids were derived from C57BL/6 mice purified alveolar type 2 (AT2) cells, as described above. Established organoids were maintained and expanded as described in Katsura et al., 2020. In short, expansion medium contained 10 µM SB431542, 3 µM CHIR99021, 1 µM BIRB796, 1 µM DMH-1, 10 µM Y-27,632 (for the first 4 days), 50 ng/mL EGF, 10 ng/mL FGF10, 10 ng/mL IL-1b, 10 ng/mL Noggin, 5 µg/mL Heparin, 1X B-27 supplement, 1X antibiotic-antimycotic, 15 mM HEPES, 1X GlutaMAX, 1.25 mM N-Acetyl-L-Cysteine. Cells were expanded in Matrigel droplets containing equal volumes Matrigel and expansion medium. Organoids were passaged every 7–10 days.

Human alveolar organoids were derived isolated AT2 cells from primary human distal lung tissue, as described above. Established organoids were maintained and expanded as described in Katsura et al., 2020. In short, expansion medium contained 10 µM SB431542, 3 µM CHIR99021, 1 µM BIRB796, 10 µM Y-27,632 (for the first 4 days), 50 ng/mL EGF, 10 ng/mL FGF10, 10 ng/mL, Heparin, 1X B-27 supplement, 1X antibiotic-antimycotic, 15 mM HEPES, 1X GlutaMAX, 1.25 mM N-Acetyl-L-Cysteine. Cells were expanded in Matrigel droplets containing equal volumes Matrigel and expansion medium. Organoids were passaged every 7–10 days for the first 5 passages.

### Alveolar organoid outgrowth efficiency assays

For the outgrowth efficiency assay murine purified primary lung AT2 cells, murine AT2 organoids cells and human AT2 organoid cells were used. Both mouse and human organoid lines were digested to small, near single-cell suspension fragments using 1X TrypLE with 10 µM Y-27,632 for 10 min at 37 °C. For both species the base treatment medium consisted of expansion medium without CHIR99021 and supplemented with 1 µM porcupine inhibitor WNT-C59 and 10 µM Y-27,632. All cells for all conditions were plated in 15 µL Matrigel droplets in round bottom 96-well plates and submerged in 120µL of the experimental medium. The medium was changed approximately every three days. Each experiment consisted of three technical replicates per plate. Outgrowth efficiency was quantified using ATP cell viability assay CellTiter-Glo measured on the SpectraMax Paradigm microplate reader (Molecular Devices) according to manufactures protocols.

### Published scRNA-seq data visualizations

Single-cell RNA sequencing (scRNA-seq) data from Haberman et al., 2020 (human lung) and Strunz et al., 2020 (mouse lung) were downloaded. The mouse dataset was filtered, removing any cell with more than 20% mitochrondrial UMIs, more than 15,000 or fewer than 300 total UMIs, or more than 4000 or fewer than 100 genes. The human dataset was filtered, removing any cell with greater than 20% mitochrondrial UMIs, fewer than 500 or more than 50,000 total UMIs, and fewer than 200 or more than 6000 genes. Scaling normalization with size factor deconvolution was applied using the *Scran* R package, and average expression per cell type was calculated using the authors’ cell type annotations and plotted using the *pheatmap* R package.

### Statistical analysis

One-way ANOVA with Holm-Sidak post-hoc test was used on all RT-qPCR data. One-way ANOVA with Fisher’s least significant difference test was applied to all lung functional data. The histology-based metrics did not appear to be normally distributed, and the non-parametric Kruskal-Wallace test followed by Dunn’s post-hoc test was applied.

### Reagent table

See Supplementary Table 3, Additional File 1.

## Results

Because Wnt signaling is critical for alveolar maintenance, we explored how treatment with WNT mimetics affected mouse and human AT2 organoids. Recently, a stroma-free expansion of adult human and mouse AT2 cells has been described; these cultures depend on Wnt signaling activation, and addition of the GSK-3 inhibitor CHIR99021 in the expansion medium is required for both mouse and human cultures [65]. We established mouse alveolospheres with purified AT2 cells from primary distal lung tissue (Fig. 1A). The purified cell population expressed the AT2 marker *Sftpc* and had no significant expression of the epithelial cell type markers *Pdpn*, *Hopx*, or *Krt5* (Fig. 1B). Mouse alveolospheres could be expanded for multiple passages and maintained a proliferative alveolar type 2 cell fate as evidenced by SFTPC and MKI67 protein levels (Fig. 1C). A WNT mimetic targeting FZD1,2,5,7,8 and LRP5,6 (L-F12578 [58]), was sufficient to replace CHIR99021 and expand the cultures (Fig. 1D). Mouse AT2 organoids were dependent on WNT ligand for growth because the cultures began to fail in the presence of the Porcupine inhibitor WNT-C59; however, at 0.1 nM L-F12578, they had similar expansion rates and cystic phenotypes to those observed with CHIR99021 (Fig. 1E, F). We applied a similar protocol to isolate and culture human AT2 alveolospheres from primary distal lung tissue. They could be expanded for several passages, maintained expression of the AT2 cell marker HTII-280, proliferated (MKI67), and phenotypically resembled those described by Katsura et al. in 2020 (Fig. 1G). In the presence of WNT-C59, L-F12578 potently stimulated expansion of human AT2 organoids *in vitro* without exogenous RSPO (Fig. 1H, I). Overall, our alveolar organoid experiments demonstrated that AT2 cells are responsive to a broad spectrum FZD-binding WNT mimetic.

The acute bleomycin model is an established model of pulmonary fibrosis with both strengths and weaknesses [66]. Because of the potentially opposing roles of active Wnt signaling, which has been shown to be important for alveolar regeneration but has also been implicated in promoting fibrosis, we investigated antibody-based positive and negative modulators of Wnt signaling in a therapeutic regimen with twice weekly dosing wherein bleomycin was administered intratracheally on day 0, and molecule treatment began on day 7 (Fig. 2A, B). We tested the following treatments: (1) a multi-FZD-specific antagonist (R2M3 which binds FZD1,2,5,7,8 [61]); (2) a combination of a WNT mimetic (R2M3-26 that binds FZD1,2,5,7,8 and LRP6 [61, 63]) plus RSPO2 [57] to activate Wnt signaling; and (3) a negative control antibody to eGFP (Anti-GFP). The combination of a WNT mimetic with RSPO2 is a way to activate the pathway regardless of whether endogenous WNT or RSPO is limiting. Mouse bodyweight was slightly greater after R2M3-26 + RSPO2 treatment than with the Anti-GFP control; however, the R2M3 antagonist treatment decreased the bodyweight (Fig. 2C), probably because of effects on the gastrointestinal tract, where Wnt signaling inhibition has been well documented to decrease epithelial maintenance [67]. Unexpectedly, the R2M3-26 + RSPO2 treatment decreased lung fibrosis, according to blinded histopathological assessments (Fig. 2D, E). Similarly, the Anti-GFP negative control and R2M3 antagonist treated samples had significantly greater ACTA2 levels (used as a proxy for myofibroblasts) than samples from saline (sham) injury animals, whereas the R2M3-26 + RSPO2 treatment samples had lower average ACTA2 levels than the negative control (Fig. 2F, G). The histological improvement encompassed reductions in inflammation, epithelial hyperplasia, and immune cell infiltration in addition to reductions in fibrosis proper (Supplementary Table 1, Additional File 1).

Because the R2M3-26 + RSPO2 treatment reduced pathology associated phenotypes, next we compared the effects of monotherapy with either a WNT mimetic (R2M3-26) or RSPO2 on fibrosis, using the same study design (Fig. 2A). In this second study, the degree of bleomycin damage was more severe, and both R2M3-26 and RSPO2 increased mouse survival and bodyweight (Fig. 3A, B). R2M3-26 significantly decreased the Modified Ashcroft Score and percentage lung fibrosis (Fig. 3D, E, G), and R2M3-26 and RSPO2 treatments showed trended decreases in the Severity Score and ACTA2 levels (Fig. 3C, F, H). This study confirmed the beneficial effects of Wnt/β-catenin pathway activation with either a broad FZD-specific WNT mimetic or the pathway enhancer, RSPO2. Untargeted RSPO treatment has potential mitogenic adverse effects in the intestines [68]; therefore, we investigated only the WNT mimetic in a subsequent study.

The effects of the R2M3-26 WNT mimetic on lung function were evaluated next in a two-hit bleomycin model with more consistent damage and fibrosis. Twice weekly treatment with either an Anti-GFP antibody or R2M3-26 started at day 10 for 2.5 weeks with termination at day 28 (Fig. 4A). R2M3-26 increased bodyweight as early as the 2-week timepoint, consistent with improved animal health (Fig. 4B). A range of functional measurements, including pulmonary elastance, static compliance (C_stat_), inspiratory capacity, expiratory time, and breathing rate, showed robust, significant improvements after R2M3-26 treatment compared with the negative control treatment (Fig. 4C-G).

R2M3-26 also led to improvements in several cell biology and histopathology metrics. The lung weight relative to bodyweight significantly improved (Fig. 4H), in agreement with less fibrosis, inflammation, and immune cell infiltration (Supplementary Table 1, Additional File 1); moreover, lung collagen content significantly decreased by day 28 (Fig. 4I). Histopathology assessment revealed a significant decrease in the Severity Score whereas the Modified Ashcroft Score and the percentage lung fibrosis trended down (Fig. 4J, K, L, Supplementary Table 1, Additional File 1). ACTA2 levels significantly decreased by day 28 (Fig. 4M, N). Furthermore, R2M3-26 treatment significantly diminished many inflammatory cytokine levels (Supplementary Table 2, Additional File 1).

Many cells express WNT receptors and could be responsive to R2M3-26, which binds FZD1,2,5,7,8. In mice, *Fzd1, Fzd2*, or *Fzd7* are enriched in fibroblasts and mesothelial cells; *Fzd1* and *Fzd7* are also detectable in several immune cells. *Fzd5* is the most specific to alveolar cells, but *Fzd2* and *Fzd7* are also expressed there to varying degrees (Fig. 4O, P). These patterns suggested that R2M3-26 might potentially act on alveolar, stromal, and immune cells. However, mRNA expression does not necessarily correspond to cell surface protein levels. To better understand the responsive cell types, we examined the acute bleomycin model (Fig. 2A) in short intervals after a single treatment at day 7. There was a significant increase in expression of the Wnt/β-catenin target gene *Axin2* in the lungs after R2M3-26 treatment (Fig. 4Q). *Axin2* expression was elevated at 8-hours after treatment in AT2 (SFTPC+) cells and, to an apparently greater extent, in other (non-AT2) distal lung cells (Fig. 4R). This suggests that the therapeutic effect may at least in part be mediated by impacts on cells other than AT2 cells, and future efforts will focus on understanding how the responding cells are reducing fibrosis.

## Discussion

Repeated dosing with the R2M3-26 WNT mimetic agonist with broad FZD specificity (FZD1,2,5,7,8) decreased pulmonary inflammation and fibrosis and improved lung function in the acute bleomycin model. This treatment activated Wnt/β-catenin signaling in multiple cell types, and its effects on other cell types/states beyond alveolar cells may contribute to its therapeutic benefits.

The evidence that Wnt/β-catenin pathway activation in multiple lung cell types could limit fibrosis runs counter to findings from several studies in which Wnt/β-catenin activation promoted or was associated with lung fibrosis [41–49, 52–54]. Our results were initially unexpected because we assumed that pathway activation targeted to the alveolar epithelium or broader (non-alveolar targeted) pathway antagonism would be necessary to achieve efficacy. Some potential explanations are that any negative (pro-fibrotic) effects on myofibroblasts might have been overridden by effects on epithelial, other stromal, and/or immune cells and that ligand mimetic induced Wnt/β-catenin signaling might not necessarily promote TGFβ-driven fibrosis. Ultimately, our findings highlight that the functional capabilities of WNT mimetics cannot be predicted from antagonist and knockdown/out studies but must be defined empirically with those molecules. Future work will address questions such as whether one key cell type or state plays a dominant role or the response of several in concert facilitates repair. Moreover, understanding exactly which cells are responsible for efficacy opens the door to developing therapeutics that target Wnt pathway activation specifically to those cells, limiting potential side-effects.

In pulmonary fibrosis, multiple cell types may need to be targeted to provide a regenerative environment facilitating repair: targeting Wnt/β-catenin activation predominantly to AT2 cells may not be sufficient to decrease fibrosis and restore alveolar architecture and function. In fact, in recent work, treatment with FZD5- (or FZD6-) selective mimetics designed to preferentially target alveolar epithelial cells and dosed beginning at day 0 with bleomycin led to expansion of AT2 cells into the airways without reducing fibrosis [69]. In accordance with the potential benefits of broader pathway activation, Sung and colleagues have recently shown that enhancing Wnt/β-catenin signaling by administering therapeutic dosing of an antibody targeting DKK1, an LRP receptor antagonist, limits inflammation and fibrosis in the acute bleomycin model [70]. Furthermore, one effect of R2M3-26 treatment is to decrease inflammation, and systemic administration of exogenous RSPO3 has been found to have an anti-inflammatory effect on macrophages in a series of mouse lung damage models [71]. Finally, although no published data have indicated how impacting different WNT receptors on the same cell type might affect Wnt/β-catenin pathway activation and cellular responses, such phenomena or the degree of pathway activation (the potency of the molecules) might potentially contribute to the differences in effects observed between a FZD1,2,5,7,8-specific and a FZD5-specific WNT mimetic.

## Conclusions

We show that a WNT mimetic with broad spectrum FZD specificity decreased pulmonary fibrosis and inflammation and improved lung function in the acute bleomycin model, and it increased Wnt pathway activation in multiple cell types. A broad spectrum WNT mimetic also robustly expanded alveolar organoids. Our findings suggest there is great potential for modulating Wnt/β-catenin signaling to treat lung diseases including IPF—a possibility warranting more thorough exploration. As we refine understanding of the responsive cell types, we will construct WNT and RSPO mimetics that target epitopes on select individual or groups of cell types [57, 72] with the goal of developing therapies that repair pulmonary fibrosis with limited systemic liabilities.

### Electronic supplementary material

Below is the link to the electronic supplementary material.

Supplementary Material 1

## Data Availability

The datasets used during the current study are included in the article, and the scRNA-seq dataset accession numbers are available in the original publications. Instructions for producing WNT mimetics are publicly available [73].

## Abbreviations

ACTA2: actin alpha 2 (smooth muscle actin)
ANOVA: analysis of variance
AT1: alveolar epithelial type I cell
AT2: alveolar epithelial type II cell
ATP: adenosine triphosphate
BAL: bronchoalveolar lavage
BALF: bronchoalveolar lavage fluid
DAPI: 4’,6-diamidino-2-phenylindole
ECM: extracellular matrix
EGF: epidermal growth factor
FGF: fibroblast growth factor
FISH: fluorescent mRNA in situ hybridization
Fzd: Frizzled
GFP: green fluorescent protein
H&E: hematoxylin and eosin
HEPES: 4-(2-hydroxyethyl)-1-piperazineethanesulfonic acid
IF: immunofluorescence
IPF: idiopathic pulmonary fibrosis
NBF: neutral buffered formalin
PBS: phosphate buffered saline
RT-qPCR: reverse transcriptase quantitative polymerase chain reaction
RSPO: R-spondin
scRNA-seq: single-cell RNA sequencing
TGFβ: transforming growth factor beta
UMI: unique molecular identifier
Wnt: Wingless-related integration site
