# Supplementary material for: A WNT mimetic with broad spectrum FZD-specificity decreases fibrosis and improves function in a pulmonary damage model
Source: Respir Res. 2024 Apr 2;25:153. doi: 10.1186/s12931-024-02786-2 (PMC10985870; doi:10.1186/s12931-024-02786-2)
Supplement: Supplementary file 1 — Supplementary Material 1 [file 12931_2024_2786_MOESM1_ESM.docx]

Supplementary Table 1. Histopathological observations in three bleomycin-induced lung fibrosis studies


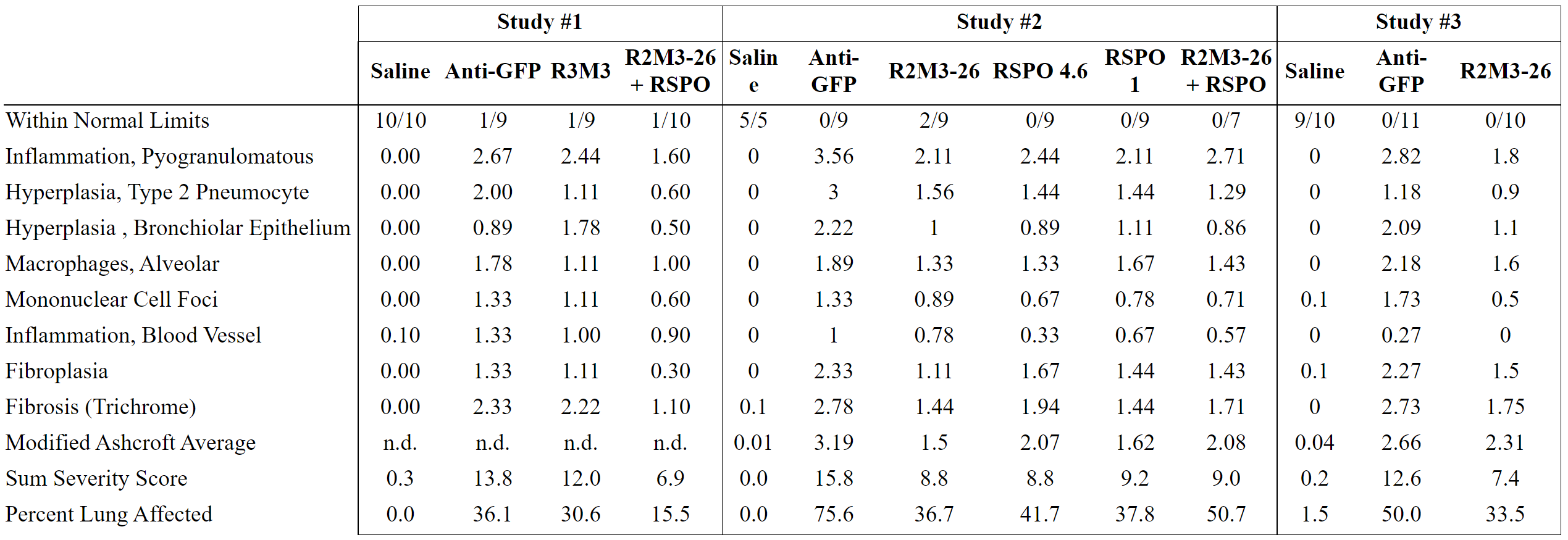


Supplementary Table 1. Histopathological observations in three bleomycin-induced lung fibrosis studies. Animals were euthanized at termination. Samples were processed as described in the Methods, and scored in a blinded manner by a veterinary histopathologist.

Supplementary Table 2. Effect of R2M3-26 on cytokines and BALF in a bleomycin-induced lung fibrosis model.

|  | **Saline** | | **Anti-GFP** | | | **R2M3-26** | | |
| --- | --- | --- | --- | --- | --- | --- | --- | --- |
|  | Average | SEM | Average | SEM | vs Saline | Average | SEM | vs anti-GFP |
| **Cytokines** | pg/mL | | | | | | | |
| CCL2 | 6.2 | *0.5* | 99.5 | *29.5* | **** | 13.7 | *1.9* | * |
| CCL3 | 5.6 | *0.9* | 19.1 | *7.7* | ** | 6.8 | *1.4* | * |
| CCL21 | 337.7 | *33.7* | 5601.5 | *1725.2* | **** | 3481.5 | *997.4* |  |
| CSF3 | 2.0 | *0.2* | 216.2 | *177.0* | **** | 9.0 | *0.7* | * |
| IFNB1 | 34.6 | *1.7* | 60.1 | *5.2* | *** | 46.3 | *4.3* |  |
| IL1A | 26.3 | *2.9* | 14.2 | *1.6* | ** | 15.0 | *2.8* |  |
| IL5 | 0.2 | *0.2* | 32.1 | *26.2* | **** | 0.9 | *0.3* | * |
| IL6 | 0.3 | *0.0* | 1367.6 | *729.7* | **** | 5.0 | *2.1* | * |
| IL11 | 2.0 | *0.3* | 170.3 | *153.1* | ** | 2.5 | *0.5* | * |
| IL16 | 349.5 | *39.8* | 1151.4 | *172.6* | *** | 747.8 | *147.7* |  |
| LIF | 0.2 | *0.2* | 27.3 | *16.7* | **** | 2.5 | *0.4* | * |
| MCP1 | 5.6 | *1.1* | 21.3 | *7.8* |  | 7.7 | *1.4* |  |
| MCP5 | 2.5 | *0.2* | 33.6 | *10.2* | *** | 25.8 | *4.2* |  |
| MDC | 17.3 | *1.3* | 55.5 | *6.7* | *** | 48.9 | *6.1* |  |
| MIP3B | 4.7 | *1.7* | 16.2 | *2.6* | ** | 12.3 | *2.8* |  |
| TARC | 15.3 | *1.5* | 98.3 | *15.8* | **** | 52.1 | *7.3* |  |
| TIMP1 | 41.8 | *4.7* | 1160.6 | *449.3* | **** | 262.2 | *43.7* | * |
| **BALF cells** | 10E9/L | | | | | | | |
| Total cell count | 0.23 | *0.02* | 0.75 | *0.20* | ****** | 0.49 | *0.40* |  |
| Macrophages | 0.22 | 0.02 | 0.39 | 0.04 | **** | 0.35 | 0.02 |  |
| Lymphocytes | 0.00 | 0.00 | 0.35 | *0.18* | **** | 0.13 | 0.02 |  |
| Neutrophils | 0.000 | 0.00 | 0.015 | 0.008 | * | 0.002 | 0.001 |  |

Supplementary Table 2. Effect of R2M3-26 on cytokines and cell numbers in the BALF in a bleomycin-induced lung fibrosis model. Cytokine levels are shown in pg/mL of BALF. Cells are shown as a proportion of 10E9/L. Asterisks represent the significance of the p-values for the indicated comparison at the top of each section as follows: * < 0.05, ** < 0.01, *** < 0.001, **** < 0.0001.

Supplementary Table 3

*Reagents*

| **REAGENT or RESOURCE** | **SOURCE** | **IDENTIFIER** |
| --- | --- | --- |
| R2M3-26 | Surrozen, Inc. | (63, 65) |
| R2M3 | Surrozen, Inc. | (65) |
| RSPO2 (RSPO2 appended to human Fc) | Surrozen, Inc. | (59) |
| Anti-GFP human IgG1 | Surrozen, Inc. | (65) |
| Advanced DMEM | Thermo Scientific | 12634-010 |
| HEPES | Thermo Scientific | 15630080 |
| GlutaMAX | Thermo Scientific | 35050061 |
| Penicillin-Streptomycin | Thermo Scientific | 15140122 |
| B27 | Thermo Scientific | 17504044 |
| N-acetylcysteine | Sigma-Aldrich | A9165 |
| CHIR99021 | Tocris | 4423 |
| WNT-C59 | Tocris | 5148 |
| SB431542 | Abcam | Ab120163 |
| BIRB796 | Tocris | 5989 |
| DMH-1 | Tocris | 41-261-0 |
| Heparin | Sigma-Aldrich | H3149 |
| Recombinant Human EGF | Peprotech | AF-100-15 |
| Recombinant Human FGF10 | Peprotech | 100-26 |
| Recombinant Human Noggin | Peprotech | 120-10C |
| Mouse IL-1ß | BioLegend | 575104 |
| Matrigel™GFR Membrane Matrix | Corning | CB40230C |
| L-F12578 | Surrozen, Inc. | PCT WO2021/173726, (60) |
| Direct-Zol RNA Microprep kit | Zymo Research | R2660 |
| Large Capacity cDNA Synthesis Kit | Applied Biosystems | 2616251 |
| Phosphate Buffered Saline without Calcium, Magnesium | Gibco | 20012-027 |
| Fetal Bovine Serum | Gibco | 16000-044 |
| Antibiotic-Antimycotic | Gibco | 15240-062 |
| Serum Free Protein Block | Agilent | X090930-2 |
| Tween-20 | Thermofisher | J-20605-AP |
| Alcohol, 70%, HistoPrep | Thermo Fisher Scientific | HC-1000-1GL |
| Formalin solution, neutral buffered, 10% | Sigma Aldrich | HT501128-4L |
| ACK Lysis Buffer | Gibco | A10492-01 |
| Liberase DL | Roche | 5401160001 |
| Dispase II | Roche | 4942078001 |
| DNAse1 | Sigma-Aldrich | 4716728001 |
| Mouse Anti-ACTA2 Antibody | Abcam | Ab7817 |
| Rabbit Anti-SFTPC Antibody | Invitrogen | PA5-71680 |
| Rat Anti-RAGE/AGER1 Antibody | R & D Systems | MAB1179 |
| HTII-280 Antibody | Terrace Bio | TB-27AHT2-280 |
| Anti-Human EPCAM Antibody, Alexa-488 | Biolegend | 324208 |
| Anti-Mouse EPCAM Antibody, Alexa-647 | Biolegend | 118212 |
| Lysotracker DND Red | Thermofisher | L7528 |
| Goat anti-mouse IgM, Alexa-647 | Thermofisher | A21238 |
| Human Fc receptor block | Miltenyl Biotec | 130-059-901 |
| Mouse Fc receptor block | Miltenyl Biotec | 130-092-575 |
| Donkey anti-rabbit IgG, highly cross adsorbed secondary antibody, Alexa Fluor 647 | Thermofisher | A31573 |
| Donkey anti-mouse IgG, highly cross adsorbed secondary antibody, Alexa Fluor 488 | Thermofisher | A21202 |
| Donkey anti-rat IgG, highly cross adsorbed secondary antibody, Alexa Fluor 568 | Thermofisher | A78946 |
| Alcohol, 70%, HistoPrep | Thermo Fisher Scientific | HC-1000-1GL |
| Formalin solution, neutral buffered, 10% | Sigma Aldrich | HT501128-4L |
| *Axin2* Mouse C1 probe | Advanced Cell Diagnostics Bio | ACD400331 |
| RNAscope Multiplex Fluorescent Reagent Kit, v2 Assay | ACD Bio | ACD323100 |
| Vectashield Vibrance antifade mounting medium with DAPI | Vector Laboratories | H-1800 |
| Tyramide Signal Amplification Plus Cyanine 3 System | Akoya Biosciences | NEL744001KT |
| Sircol™ - (Original) Soluble Collagen Assay kit | Biocolor Life Science Assays | S1111 |
| MagMAX™ mirVana™ Total RNA Isolation Kit | Thermo Fisher Scientific | A27828 |
| SuperScript™ VILO™ Master Mix | Thermo Fisher Scientific | 11755050 |
| high-Capacity cDNA Reverse Transcription Kit | Thermo Fisher Scientific | 43-688-14 |
| TaqMan® Fast Advanced Master Mix | Thermo Fisher Scientific | 4444963 |
| TaqMan® mouse Actb Mm02619580_gl | Thermo Fisher Scientific | 4351370 |
| TaqMan® mouse *Gapdh* Mm99999915_g1 | Thermo Fisher Scientific | 4331182 |
| TaqMan® mouse *Sftpc* Mm00488144_m1 | Thermo Fisher Scientific | 4331182 |
| TaqMan® mouse *Krt5* Mm00503549_m1 | Thermo Fisher Scientific | 4331182 |
| TaqMan® mouse *Pdpn* Mm01348912_g1 | Thermo Fisher Scientific | 4331182 |
| TaqMan® mouse *Hopx* Mm00558630_m1 | Thermo Fisher Scientific | 4331182 |
| TaqMan® mouse *Axin2* Mm00443610_m1 | Thermo Fisher Scientific | 4331182 |
